# Why does asking questions change health behaviours? The mediating role of attitude accessibility

**DOI:** 10.1080/08870446.2013.858343

**Published:** 2013-11-19

**Authors:** Chantelle Wood, Mark Conner, Tracy Sandberg, Gaston Godin, Paschal Sheeran

**Affiliations:** a Department of Psychology, University of Sheffield, Sheffield, UK; b Institute of Psychological Sciences, University of Leeds, Leeds, UK; c Faculty of Nursing, Research Group on Behaviours and Health, Laval University, Québec City, Canada

**Keywords:** question-behaviour effect, mere-measurement, attitude accessibility, intentions, behaviour change, healthy eating

## Abstract

**Objective:**

The question-behaviour effect (QBE) refers to the finding that measuring behavioural intentions increases performance of the relevant behaviour. This effect has been used to change health behaviours. The present research asks why the QBE occurs and evaluates one possible mediator – attitude accessibility.

**Design:**

University staff and students (*N* = 151) were randomly assigned to an intention measurement condition where they reported their intentions to eat healthy foods, or to one of two control conditions.

**Main outcome measures:**

Participants completed a response latency measure of attitude accessibility, before healthy eating behaviour was assessed unobtrusively using an objective measure of snacking.

**Results:**

Intention measurement participants exhibited more accessible attitudes towards healthy foods, and were more likely to choose a healthy snack, relative to control participants. Furthermore, attitude accessibility mediated the relationship between intention measurement and behaviour.

**Conclusion:**

This research demonstrates that increased attitude accessibility may explain the QBE, extending the findings of previous research to the domain of health behaviour.

A major goal of research in health psychology is to identify ways in which we can promote desirable changes in behaviour, and to understand the mechanisms that underlie these changes. One simple but effective means to change health behaviour is to ask people questions about a behaviour (e.g. their intentions), as doing so influences the likelihood and rates of performing that behaviour (Dholakia, 2010). This phenomenon goes by a variety of terms including the question-behaviour effect (QBE), the mere-measurement effect, self-erasing errors of prediction, the self-prophecy effect and self-generated validity (Sprott, Spangenberg, Block et al., 2006). Although the QBE has been replicated empirically in several behavioural domains, there is less direct evidence concerning the mechanisms that underlie the effect, particularly in the domain of health behaviour.

The QBE has been successfully observed for a variety of health behaviours including healthy eating (Levav & Fizsimons, 2006; Williams, Fitzsimons, & Block, 2004), health club attendance and exercise (Godin, Bélanger-Gravel, Amireault, Vohl, & Perusse, 2011; Sandberg & Conner, 2011; Spangenberg, 1997; Spangenberg, Sprott, Grohmann, & Smith, 2003; Spence, Burgess, Rodgers, & Murray, 2009; Williams, Block, & Fitzsimons, 2006), blood donation (Cioffi & Garner, 1998; Godin, Germain, Conner, Delage, & Sheeran, in press; Godin, Sheeran, Conner, & Germain, 2008), and uptake of health assessments, screening and vaccinations (Conner, Godin, Norman, & Sheeran, 2011; Sandberg & Conner, 2009; Spangenberg & Sprott, 2006; Sprott, Smith, Spangenberg, & Freson, 2004; Sprott, Spangenberg, & Fisher, 2003), though the QBE has not always proved reliable (Ayres et al., 2013; Godin et al., 2010; van Dongen, Abraham, Ruiter, & Veldhuizen, 2013). A meta-analysis of the impact of the QBE on health behaviour change revealed a small-to-medium effect size of *z*_r_ = .265 (Sprott, Spangenberg, Knuff et al., 2006). This effect size is comparable in magnitude to the impact of more complex manipulations of intentions in promoting health behaviours (e.g. see Webb & Sheeran, 2006, for a meta-analysis), suggesting that particularly in the health domain, strategic use of the QBE could form a simple and cost-effective method to change behaviour.

## What drives the question-behaviour effect?

While measurement of cognitions such as intentions appears to influence behaviour, research in this field has only relatively recently focused on identifying the mechanisms underlying this effect. Indeed, the overwhelming focus of existing research has been on demonstrating the existence and scope of the QBE rather than the processes driving it, as noted by others (e.g. Chapman, 2001; Spangenberg et al., 2003). However, a good understanding of the mechanisms responsible for behaviour change due to intention measurement is critical to harnessing the QBE to best effect.

One key explanation for the QBE is that asking questions increases the accessibility of attitudes towards the behaviour, which in turn makes it more likely that the person will perform that behaviour (Dholakia, 2010). Attitude accessibility refers to the strength of the association in memory between mental representations of a behaviour and its evaluation (Fazio, 1990b; Fazio & Towles-Schwen, 1999), and is characteristically measured by how quickly participants respond to queries about the attitude. According to this explanation of the QBE, questions about intentions or related cognitions serve to activate (i.e. render more accessible in memory) the underlying attitude to the behaviour. If participants are not asked to express their intentions, then the relevant attitude may not be spontaneously retrieved or generated, and so this attitude should be less influential in guiding behaviour (Feldman & Lynch, 1988). There is considerable evidence that accessibility determines the likelihood that attitudes are translated into action. For instance, meta-analysis has indicated that the attitude–behaviour relation is significantly stronger when attitudes are more accessible (*r* = .61) as compared to less accessible (*r* = .50, *p* < .05) (Cooke & Sheeran, 2004).

Attitude accessibility explanations of the QBE find some support in previous research. A number of studies in the consumer domain have evaluated this explanation by deliberately manipulating the accessibility of attitudes towards different consumer choice options (e.g. novel candy bars) and examining the impact on subsequent choice behaviour. Consistent with an attitude accessibility explanation, studies have demonstrated that participants who were asked about their intentions were more likely to choose the positive accessible target option, relative to control participants who were not asked about their intentions (Fitzsimons & Williams, 2000, Experiments 1 and 2; Morwitz & Fitzsimons, 2004, Experiment 1). Similar patterns of choice behaviour have also been demonstrated for consumer items with disproportionately accessible attitudes due to previous purchase experience (Chandon, Morwitz, & Reinartz, 2004).

Although these studies are consistent with an attitude accessibility explanation of the QBE, it is notable that none of the studies examined the effect of questioning on attitude accessibility directly. In order to provide unequivocal evidence for the role of attitude accessibility in the QBE, however, it is essential to formally measure this mechanism. To date, only a few studies have sought to evaluate the attitude accessibility explanation for the QBE using a direct measure of accessibility. In one such study, Chapman (2001) had participants rate their purchase intentions and attitudes towards a set of fictitious products in a computerised accessibility task, in which response latencies to the attitude items formed the measure of attitude accessibility. Consistent with an attitude accessibility explanation for the QBE, participants who had reported their purchase intentions prior to the task exhibited more accessible attitudes and were subsequently more likely to indicate a preference for the target products than participants who had not been asked about their intentions. However, the inclusion of intention questions in the accessibility task brings into question the adequacy of the ‘no intention’ control in this study, as all participants were required to report their purchase intentions. This issue therefore limits the degree to which this research permits any firm conclusions.

Unlike Chapman (2001), research conducted by Morwitz and Fitzsimons (2004, Experiment 3) included a true no-intention control group. Morwitz and Fitzsimons had half their participant sample make good/bad judgements about a set of novel candy bars with latencies to the target candy forming the measure of attitude accessibility. The other half of the sample was instead asked to choose which of the candy bars they would like to receive. Demonstrating the QBE, Morwitz and Fitzsimons found that participants that had been previously asked about their purchase intentions were significantly more likely to choose the target candy than participants who had not been asked about their intentions. Furthermore, consistent with an attitude accessibility explanation, participants asked about their purchase intentions also exhibited more accessible attitudes than participants who had not been asked about their intentions. However, although these findings are consistent with an attitude accessibility explanation, the fact that attitude accessibility and behaviour were measured in two separate conditions means that there was no direct statistical test of the mediating effect of attitude accessibility on the relationship between intention measurement and behaviour. That is, the study design precluded a test of the simultaneous relationship between all three variables, and whether increased attitude accessibility actually predicted target candy bar choice.

## The present study

The aim of the present study was to test formally whether attitude accessibility explains the impact of measuring intentions on subsequent health behaviour, and thus to examine whether previous findings supporting the role of attitude accessibility in consumer behaviours can be extended to the health domain. Healthy eating behaviour was investigated because disease stemming from poor diet is the second most common cause of death in the USA (Mokdad, Marks, Stroup, & Gerberding, 2004) and has a larger cost burden on the National Health Service (NHS) in the UK than any other lifestyle factor (Scarborough et al., 2011). The test involved comparing a condition that measured behavioural intentions and other cognitions relating to eating healthily with two control conditions (an alternative intention condition and an irrelevant task condition). The accessibility of attitudes to healthy food was measured using a response latency task in all conditions. We also included an unobtrusive, objective measure of healthy vs. unhealthy food consumption in the wake of the manipulations and accessibility task. The predictions tested were that (a) measuring intentions will increase healthy food consumption, (b) measuring intentions will heighten the accessibility of attitudes to healthy food and (c) attitude accessibility will mediate the relationship between intention measurement and food consumption.

## Method

### Design

This study had a hierarchical between-subjects design with one factor (Condition: Healthy eating intentions vs. control) and two nested levels for the control group (Internet use intentions vs. word jumble). Both unrelated-intention (internet use) and no-intention (word jumble) control groups were included as both types of control group have been used in previous question-behaviour research, and we wished to provide a stern test of the QBE. Ethical approval for the study was given by a University in the north of England where the research was conducted. The study was conducted in line with these ethical guidelines and those of the British Psychological Society.

### Participants

Participants were 151 student and staff volunteers from a variety of departments and study areas at a University in the north of England, who were paid £5. Participants (111 females, 40 males) had a mean age of 24.48 years (SD = 8.67); 76.82% of the participants were undergraduate or postgraduate students. Allocation to conditions was counterbalanced using the stimuli presentation software E-Prime Version 1.2 (Schneider, Eschman, & Zuccolotto, 2002). There were approximately equal numbers of participants in the healthy eating intentions (experimental) condition (*n* = 51; 37 female, 14 male), the internet use intentions (control) condition (*n* = 50; 37 female, 13 male) and the word jumble (control) condition (*n* = 50; 37 female, 13 male).

### Measures and procedure

#### Intention measurement manipulation

E-Prime Version 1.2 (Schneider et al., 2002) was used to present stimuli and record responses. After signing consent forms, participants in the two intention conditions were first presented with a series of questionnaire items on a computer screen and asked to respond to each item by pressing a number from 1 to 7 on the keyboard. Responses were recorded by the computer. For participants assigned to the healthy eating intentions condition, the questionnaire was a standard Theory of Planned Behaviour questionnaire modelled on Sandberg and Conner (2009, 2011) comprising nine questions relating to eating healthy food. Three items measuring anticipated regret were presented first (‘If I did not eat healthy foods in the next few weeks I would feel regret’, Definitely No/Definitely Yes; ‘If I imagine that I did not eat healthy foods in the next few weeks, I would feel regret’, Definitely No/Definitely Yes; ‘If I did not eat healthy foods in the next few weeks, I would later wish I had’, Strongly Disagree/Strongly Agree), followed by the critical three intention items ('I will eat healthy foods in the next few weeks’, Strongly Disagree/Strongly Agree; ‘My intention to eat healthy foods in the next few weeks is …’, Not at all Strong/Strong; ‘I want to eat healthy foods in the next few weeks’, Strongly Disagree/Strongly Agree). Finally, three items measuring attitudes were presented (‘For me, eating healthy foods in the next few weeks would be …’, Not Worthwhile/Worthwhile; Bad/Good; Not Beneficial/Beneficial). Each intention item was scored such that higher scores indicated a greater level of intention, and the scale proved reliable (*a* = .89, MIC = .74). The anticipated regret and attitude scales had good and moderate reliability, respectively (*a* = .86, MIC = .67 and *a* = .65, MIC = .39).

For participants assigned to the internet use intentions control condition, the questionnaire consisted of nine questions similar to above, but with the critical intention and other items instead relating to use of the internet (i.e. ‘I will use the internet in the next few weeks’, Strongly Disagree/Strongly Agree; ‘My intention to use the internet in the next few weeks is …’, Not at all Strong/Strong; ‘I want to use the internet in the next few weeks’, Strongly Disagree/Strongly Agree).

Participants assigned to the word jumble control condition were presented with a 9 × 9 cell letter grid containing hidden words unrelated to food (e.g. turtle), and asked to locate as many words as they could in 1 min and type each word into the computer.

#### Attitude accessibility measure

All participants then completed an attitude accessibility task. On each trial in this task, a fixation cross was presented in the centre of the computer screen for 1 s, followed by a target word. Participants were instructed to indicate their judgment of the word, as quickly as possible, by pressing the ‘good’ or ‘bad’ key on the keyboard. The key assigned to good and bad (m and z) was counterbalanced for each participant. Target words remained on screen until a response was made, followed by an inter-trial interval of 1 s. Responses and response latencies were recorded.

There were 10 practice trials followed by 160 experimental trials. Practice trials presented stimuli unrelated to food (e.g. slippers, radio). The critical 20 experimental trials consisted of 10 trials presenting healthy food stimuli (e.g. apple, broccoli) and 10 trials presenting unhealthy food stimuli (e.g. cake, chips). These two categories were chosen as they map on to the healthy vs. unhealthy food choice task that forms the key measure of behaviour (discussed in detail later). An additional 10 trials presented filler health-neutral food stimuli (e.g. beef, eggs) to mask the focus on healthy vs. unhealthy food items. See Appendix 1 for a full list. Experimental stimuli were selected from a larger pool of food words that had been pilot tested on healthiness. Twenty-two participants independent of the main study (16 female and 8 male, mean age = 33.4) rated 64 different foods on their perceived healthiness using a seven-point scale (where 1 = Very unhealthy and 7 = Very healthy). The selected three sets of words differed significantly in their perceived healthiness, *F*(2, 27) = 518.00, *p* < .001, *ηp*^2^ = .97, such that the healthy food stimuli had the highest healthiness rating (*M* = 6.41, SD = .21) and the unhealthy food stimuli the lowest (*M* = 1.78, SD = .31), with the neutral food stimuli characterised by a mean healthiness rating around the midpoint of the scale (*M* = 4.26, SD = .43). The word sets did not differ in word length, *F*(2, 27) = .56, *p* = .57, *ηp*^2^ = .04, or Kucera Francis word frequency, *F*(2, 27) = .92, *p* = .41, *ηp*^2^ = .06. The remaining 130 trials presented neutral filler stimuli unrelated to food (e.g. winter, rollerskates). Order of presentation of stimuli was randomised for each participant.

#### Filler task

Participants then completed the Stroop test (Stroop, 1935) as a filler task designed to reduce suspicion regarding the relationship between the questionnaire and the behavioural measure. On each trial in this task, participants were presented with a fixation cross followed by a colour-name word which was presented in either a congruent colour font (i.e. the word blue in blue font) or an incongruent colour font (i.e. the word blue in red font). Participants were instructed to indicate the font colour, as quickly but as accurately as possible, by pressing one assigned key if the colour of the font was green or red, and another assigned key if the colour of the font was blue or yellow. Participants completed 70 trials in total.

#### Healthy eating behaviour measure

Healthy eating behaviour was measured using a food choice paradigm. At the conclusion of the experiment, participants were offered a snack choice from two bowls in an adjoining room, ostensibly as a thank you for participating. One bowl was filled with 15 ‘healthy’ fruit items (apples, bananas, pears, satsumas and nectarines) and the other with 15 ‘unhealthy’ chocolate bars (Dairy Milk, Twix, Twirl, Mars, Flake). The adjoining room was located such that participants had to walk through it to exit the experiment, with their food choice made out of view of the experimenter. After the participant had left, the experimenter determined the food choice(s) made by counting the number of items left in each box. Finally, participants were sent three questions via email to assess suspicion of the experiment's purpose (What did you think the study was about?; Were you suspicious at any point that the study was looking at something other than what was stated?; If you answered “yes” to the above, what in particular were you suspicious about?), before being sent a written debriefing sheet.

## Results

### Manipulation checks

Previous research has demonstrated that the QBE is attenuated when participants suspect that measurement of intentions is intended to manipulate or change behaviour (Williams et al., 2004). To evaluate the possibility that suspicion regarding behavioural observation may affect behaviour, participants’ reports of suspicion and answers regarding the experiment's purpose that were obtained by email were coded into three levels. Suspicion was coded as 2 if participants correctly reported suspicions that food choice was observed, 1 if participants indicated more vague suspicions regarding a possible focus on healthy eating and 0 if participants indicated no suspicion at all. Twenty-six participants gave no response to the suspicion questions and were not included in this manipulation check. An ANOVA conducted on the healthy eating behaviour data revealed no significant differences between participants expressing correct suspicions (*M* = .29, SD = .41, *n* = 21), more vague suspicions (*M* = .45, SD = .47, *n* = 11) or no suspicion (*M* = .26, SD = .36, *n* = 67) on the proportion of healthy food items chosen, *F*(2, 96) = 1.21, *p* = .30, *ηp*^2^ = .02. Accordingly, suspicion was not included as a factor in subsequent analyses.

### Data screening

#### Behavioural data

Twenty-four participants (16% of the sample) did not select any food at the conclusion of the experiment and were excluded from further analysis – due to the ambiguity in classifying the absence of food choice as either healthy or unhealthy. Excluded participants were evenly distributed across conditions. Of the remaining 127 participants, 39 (26% of the sample) selected more than one food item and 29 out of these 39 participants made selections from both bowls (condition did not influence these selections). While inclusion of the 29 participants precludes a simple dichotomous categorisation of food choice as healthy or unhealthy, the loss of data that would result from excluding these participants was considered unacceptable. Accordingly, the full sample of 127 participants was utilised and the proportion of healthy items chosen formed the dependent measure of healthy eating behaviour. Higher scores indicate a greater proportion of healthy choices.

#### Attitude accessibility data

As Fazio (1990a) recommended, response latencies less than 200 ms or more than three standard deviations above each condition mean were removed from the attitude accessibility data (302 latencies in total representing 1.49% of responses). In order to ensure that the attitude accessibility measure reflects attitudes towards healthy foods specifically, rather than food more generally, attitude accessibility difference scores were calculated by subtracting the mean response latency to unhealthy stimuli from the mean response latency to healthy stimuli, such that a lower score indicates greater relative accessibility of attitudes towards healthy foods. This formed the key dependent measure of attitude accessibility. Participants with an attitude accessibility difference score greater than three standard deviations above or below the overall mean (*n* = 2) were excluded from further analysis (Fazio, 1990a).

Accordingly, data from 125 participants (41, 42 and 42 participants from the healthy eating intentions, internet use intentions and word jumble conditions, respectively) were used in the analysis of behavioural and attitude accessibility results (83% of original sample).

### Effects on behaviour

**Figure 1.**
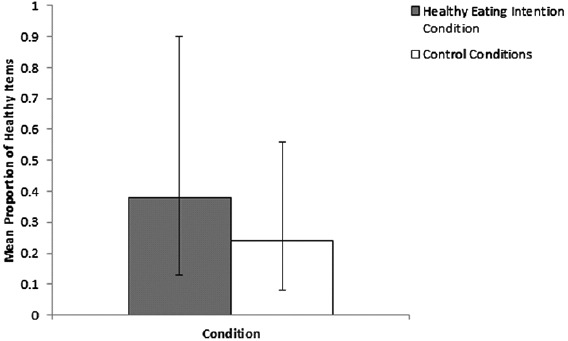
Mean proportion of healthy items in total items chosen for healthy eating intentions and control conditions, with 95% confidence intervals.

Eating behaviour data were subjected to a hierarchical ANOVA with one factor (Condition: Healthy eating intentions vs. control) and two nested levels for the control group (Internet use intentions vs. word jumble). This analysis revealed a significant effect of condition on the proportion of healthy items in total items chosen, with a small to medium effect size, *F*(1, 122) = 4.10, *p* = .04, *ηp*^2^ = .03, but no effect of the nested levels of the control condition, *F*(1, 122) = .77, *p* = .38, *ηp*^2^ = .01. Consistent with a QBE, participants in the healthy eating intentions condition chose a greater proportion of healthy food (*M* = .38, SD = .42) relative to participants in the control conditions (*M* = .24, SD = .36) (see Figure 1).^1^ The non-significant effect of control condition indicates that there was no difference in healthy eating behaviour between participants who completed internet intention questions (*M* = .20, SD = .34) and participants who completed the word jumble (*M* = .27, SD = .38).

### Effects on attitude accessibility

**Figure 2.**
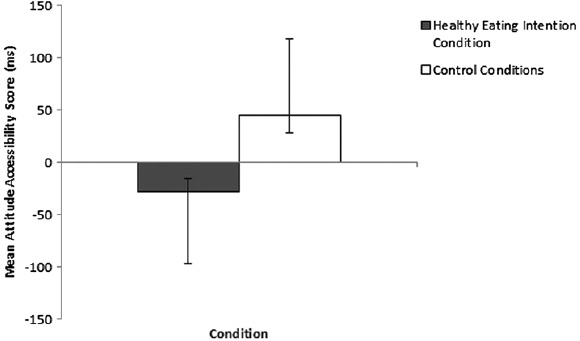
Mean attitude accessibility difference scores for healthy eating intentions and control conditions, with 95% confidence intervals.

The attitude accessibility data were subject to the same hierarchical ANOVA as above, revealing a significant effect of condition on the attitude accessibility difference score, with a medium effect size, *F*(1, 122) = 8.49, *p* = .004, *ηp*^2^ = .06, but no effect of the nested levels of control condition, *F*(1, 122) = 2.06, *p* = .15, *ηp*^2^ = .02. Consistent with predictions, participants in the healthy eating intentions condition had a lower attitude accessibility score (*M* = −28.23, SD = 129.94) indicative of more accessible attitudes to healthy foods compared to unhealthy foods, relative to participants in the control conditions (*M* = 44.99, SD = 133.69) (see Figure 2).^2^ The non-significant effect of control condition indicates that there was no difference in attitude accessibility between participants who completed internet intention questions (*M* = 24.33, SD = 133.59) and participants who completed the word jumble (*M* = 65.64, SD = 132.13).

### Mediation analysis

The correlation between the mediator (attitude accessibility) and outcome variable (healthy eating behaviour) was significant (*r* = −.20, *p* = .02); greater relative accessibility of healthy food attitudes was associated with a greater proportion of healthy foods selected. Given that the type of control group (internet intentions vs. word jumble) had no influence on behaviour or accessibility scores, the condition variable was dummy coded (experimental = 1, control = 0). The mediator (attitude accessibility score) and outcome variable (proportion of healthy items in total items chosen) were then standardised. Mediation was tested using bootstrapping analysis by means of the SPSS Mediate macro described in Hayes and Preacher (2011). Consistent with expectations, bootstrapping analysis with 5000 resamples revealed a bootstrap point estimate of .09 (SE = .06) with a percentile-based 95% confidence interval from .01 to .23.^3^ As the confidence interval does not encompass zero, these findings indicate that attitude accessibility is a significant mediator of the QBE.

## Discussion

The aim of the current research was to test whether attitude accessibility underlies the impact of intention measurement on subsequent health behaviour. First, we successfully demonstrated the QBE in our chosen health behaviour, showing that measurement of intentions to eat healthy foods resulted in a significantly greater tendency to choose a healthy snack relative to two control groups. Second, measuring intentions increased the relative accessibility of attitudes towards healthy foods, consistent with an attitude accessibility explanation for the QBE. Finally, formal mediation analyses demonstrated that the impact of answering intention questions was mediated by attitude accessibility. That is, greater relative accessibility of attitudes towards healthy foods prompted by intention measurement explained the healthier snack choice in the healthy eating intention condition compared to the control conditions.

The current study adds to the increasing pool of research that has demonstrated the impact of intention measurement on health-related behaviours (e.g. Cioffi & Garner, 1998; Conner et al., 2011; Godin et al., 2008, 2010; Levav & Fizsimons, 2006; Sandberg & Conner, 2009, 2011; Spangenberg, 1997; Spangenberg & Sprott, 2006; Spangenberg et al., 2003; Spence et al., 2009; Sprott et al., 2003, 2004; Williams et al., 2004, 2006). In doing so, it provides further support for the possibility that this effect may be harnessed as an easy and effective health behaviour change intervention.

More importantly, the present study provides a direct test of the attitude accessibility explanation for the QBE in the domain of health behaviour, supporting and extending the findings of previous research in consumer psychology. While a number of studies have supported the argument that increases in attitude accessibility drive behaviour change in the QBE (e.g. Chandon et al., 2004; Chapman, 2001; Fitzsimons & Williams, 2000; Morwitz & Fitzsimons, 2004), none have provided direct process evidence for the role of accessibility. The current work remedies this situation by employing direct measures of attitude accessibility and behaviour and demonstrating formally that attitude accessibility mediates the QBE.

As the current study is the first to directly demonstrate the key role that attitude accessibility plays in the QBE with regards to health behaviours, further research is needed to corroborate and extend these findings. For example, future research should seek to examine the time frame in which attitude accessibility operates to mediate the QBE. The present research observed healthy eating behaviour within the laboratory, demonstrating the importance of attitude accessibility in explaining behaviour performed a relatively short time after intention measurement (see Ayres et al., 2013 for a similar demonstration of the short-term impact of the QBE on health behaviour). Previous research has also demonstrated the impact of QBE on health behaviours performed after longer periods of time, with the impact extending to behaviours observed months after questioning (Conner et al., 2011; Godin et al., 2008, 2011; Williams et al., 2006). It would be valuable for future research to test precisely how durable are the increases in attitude accessibility prompted by questions about intentions.

Future research should also seek to examine other potential mediators of the QBE. For instance, it has been proposed that cognitive dissonance may underlie the QBE, as intention questions could cause participants to become aware of discrepancies between their attitudes and actions (see Dholakia, 2010 for a review). However, there is currently little in the way of direct evidence for this mechanism in the QBE (e.g. Levav & Fitzsimons, 2006; Spangenberg & Sprott, 2006; Sprott et al., 2003). Accordingly, further research should seek to examine whether additional mechanisms complement the role that attitude accessibility plays in explaining the QBE, or whether dissonance actually is mediated by heightened attitude accessibility.

It should also be acknowledged that the nature of the behavioural measure used in the current study meant that data from a relatively large number of participants could not be included in analysis. As reported in the results section, a total of 16% of participants did not select any food from the snack options at the conclusion of the experiment, a behaviour that was difficult to code as either healthy or unhealthy. To avoid loss of data, future research should employ behavioural measures that circumvent this issue. It should also be noted that participants in the present study were predominantly female (74% of the original sample). As females are more likely to consume the recommended amounts of fruit and vegetables in their daily diet (NHS Information Centre, 2011), this may have led to an overall bias towards healthy snack choice (i.e. fruit) in the present study. However, as described in the method, the proportions of female participants were equal across conditions, leaving it unlikely that any gender bias in healthy snack choice could account for the results.

The involvement of attitude accessibility in driving the QBE, however, may have implications for the broader utility of the QBE as a basis for health behaviour change intervention. First, if the accessibility of existing attitudes drives behaviour change, then we may expect behaviour change to occur in a direction consistent with the associated attitude (i.e. increased behaviour in the case of positive attitudes, and decreased behaviour in the case of negative attitudes) (Conner et al., 2011; Morwitz & Fitzsimons, 2004; Sherman, 1980). Indeed, some research suggests that this is the case (Conner et al., 2011, Study 2; Morwitz & Fitzsimons, 2004, Study 1; Williams et al., 2004, Studies 1–2). Accordingly, applying the QBE in instances where people hold negative attitudes towards a healthy behaviour may actually be detrimental to performance of that behaviour (Conner et al., 2011).

Second, identifying ways in which we can strengthen the impact of questioning on attitude accessibility is essential in maximising the utility of the QBE as a health behaviour change intervention. More research is therefore needed to examine whether certain types of questions have a stronger impact on attitude accessibility, and accordingly yield stronger QBEs. While we focused on measurement of behavioural intentions in the current study (i.e. ‘My intention to eat healthy foods in the next few weeks is …’, Not at all strong/Strong), measurement of expectations regarding the likelihood of behaviour are also common in the QBE literature (e.g. ‘How likely are you to exercise in the next two months’, Definitely will/Definitely will not; Williams et al., 2006). Given evidence that attitudes play a greater role in forming intentions than in forming expectations (e.g. Sheppard, Hartwick, & Warshaw, 1988), it seems logical that intention measurement may be more likely to activate attitude representations than other question types, and may therefore have a larger impact on attitude accessibility (and subsequently, behaviour). Relatedly, recent research suggests that intention questions have a larger impact on behaviour when they are worded in their interrogative form (i.e. Will I …?) relative to the declarative form used in the present study (i.e. I will …) (Godin, Bélanger-Gravel, Vézina-Im, Amireault, & Bilodeau, 2012). However, it is as yet unknown whether these differences are driven by differential effects on attitude accessibility. Further research should therefore seek to examine the effect of different question types on attitude accessibility and subsequent behaviour.

We should also consider the possibility that the concurrent measurement of attitudes with intentions in the current study may have had undue impact on support for the role of attitude accessibility in the QBE. Measurement of attitudes along with intentions is common in question-behaviour research (e.g. Conner et al., 2011; Godin et al., 2008, 2011; Sandberg & Conner, 2009), and as a result, it is often difficult to separate the measurement effects of each type of cognition. Research that does separate out the effects of attitudes and intentions reveals mixed findings. Using a response latency paradigm, Chapman (2001) found little difference in the accessibility of attitudes towards fictitious products between participants who responded to multiple intention items relative to those who responded to multiple attitude items. This suggests that the inclusion of multiple attitude items in the present study is unlikely to have driven the attitude accessibility effects observed in the current research. However, Chapman did find some evidence of differential attitude accessibility following attitude vs. intention measurement when these constructs were assessed with a single item. While beyond the scope of this paper, evaluation of the differential impact of intention and attitude measurement on attitude accessibility within the QBE would be a valuable avenue for further research.

## Conclusion

The current research tested whether increased attitude accessibility explains the QBE. Findings demonstrated the beneficial effect of intention measurement (vs. no measurement) on health behaviour; snack choice was healthier when intentions to eat healthily were measured relative to two control conditions. Our research also indicated that relative increases in the accessibility of healthy food attitudes prompted by intention measurement drive the observed changes in behaviour. This research provides direct evidence of the mechanisms involved in the QBE in the health domain and has important implications for future research designed to maximise behaviour change using this technique.

## Appendix 1. Attitude accessibility task stimuli for experimental trials

### Healthy food stimuli

Apple

Banana

Broccoli

Carrot

Fish

Lettuce

Orange

Pear

Spinach

Tomato

### Neutral food stimuli

Beef

Eggs

Cereal

Cheese

Ham

Honey

Mustard

Pasta

Peanuts

Popcorn

### Unhealthy food stimuli

Cream

Cake

Chips

Crisps

Bacon

Lollipop

Pie

Pizza

Sweets

Toffee
